# Detection Patterns of Porcine Parvovirus (PPV) and Novel Porcine Parvoviruses 2 through 6 (PPV2–PPV6) in Polish Swine Farms

**DOI:** 10.3390/v11050474

**Published:** 2019-05-24

**Authors:** Dagmara Miłek, Aleksandra Woźniak, Magdalena Guzowska, Tomasz Stadejek

**Affiliations:** 1 Department of Pathology and Veterinary Diagnostics, Faculty of Veterinary Medicine, Warsaw University of Life Sciences, Nowoursynowska 159C, 02-776 Warsaw, Poland; dagmara_milek@sggw.pl (D.M.); aleksandra_wozniak@sggw.pl (A.W.); 2 Department of Physiological Sciences, Faculty of Veterinary Medicine, Warsaw University of Life Sciences, Nowoursynowska 159, 02-776 Warsaw, Poland; magdalena_guzowska@sggw.pl

**Keywords:** pig, oral fluid, serum, feces, piglets, weaners, fatteners, porcine parvoviruses, real-time PCR

## Abstract

Porcine parvovirus (PPV) is a major causative agent in reproductive failure, but in the last two decades many novel porcine parvoviruses were described and designated as porcine parvovirus 2 through 6 (PPV2–PPV6). However, their role for pig health is largely unknown. The aim of this study was to better understand the on-farm prevalence of PPVs in different age groups of pigs, and to assess the diagnostic applicability of testing different diagnostic materials. In total, 271 oral fluids, 1244 serum samples, and 1238 fecal samples were collected from 3–21-week-old pigs from 19 farms, and after pooling by 4–6, tested by real-time PCR. The results showed that PPVs are widely spread in Poland and that the highest detection rates were obtained for oral fluids (ranging from 10.7% (PPV1) to 48.7% (PPV2)). Fattening pigs were the age group with the most frequent detection of PPVs (ranging from 8.6% (PPV1) to 49.1% (PPV2)). Porcine parvoviruses were detected mostly in growing-finishing pigs and the infection persisted until the late fattening period, which may suggest the chronic character of the infection (especially for PPV2, which was found to commonly infect animals of all ages). Particularly low Ct values detected for PPV2, PPV3, PPV5, and PPV6 in serum pools from some farms suggested that these viruses may cause high levels of viremia in one or more individuals included in these pools. Further studies are needed to quantify the levels of PPVs viremia and to assess the impact in co-infections with other, often endemic pig viruses, such as porcine circovirus type 2 (PCV2) and porcine reproductive and respiratory syndrome virus (PRRSV).

## 1. Introduction

Parvoviruses are small, non-enveloped, single-stranded DNA viruses with a genome of 4–6.3 kb in size [1]. They are members of the *Parvoviridae* family, which includes two subfamilies: *Parvovirinae*, infecting vertebrates, and *Densovirinae*, infecting arthropods [1].

Until recently, porcine parvovirus (PPV) was the sole representative of *Parvovirinae* members infecting pigs. Porcine parvovirus causes fetal death, mummification, stillbirths, and delayed return to estrus in naïve dams [2,3]. This virus was also found to be a co-factor of post-weaning multisystemic wasting syndrome (PMWS) [4].

In the last two decades, many novel porcine parvoviruses were described [5,6,7,8,9,10,11]. The most current taxonomy proposed by the International Committee on Taxonomy of Viruses (ICTV) classified PPV into the *Protoparvovirus* genus (species *Ungulate protoparvovirus 1*), the novel viruses such as porcine parvovirus 2 (PPV2) and porcine parvovirus 3 (PPV3) into the *Tetraparvovirus* genus (species *Ungulate tetraparvovirus 3* and *Ungulate tetraparvovirus 2*, respectively), and porcine parvovirus 4 (PPV4) into the *Copiparvovirus* genus (species *Ungulate copiparvovirus 2*) [12]. Porcine parvovirus 6 (PPV6) is described by ICTV as a related, unclassified virus in the *Copiparvovirus* genus [12]. Porcine parvovirus 5 (PPV5) is still not classified in the ICTV taxonomy proposal, but genetic analysis showed that this virus is most closely related to PPV4 [9]. The most recently discovered porcine parvovirus 7 (PPV7) is also unclassified, but in contrast to PPV5, it is only distantly related to the other porcine parvoviruses, and for this virus, the new genus *Chappaparvovirus* was proposed [11]. For the readers’ convenience, PPV has been provisionally named PPV1 in this paper.

Different studies have revealed very wide geographical distribution of novel porcine parvoviruses. All of the PPV species were detected in China, the USA, and Poland [10,11,13,14,15,16,17,18,19,20]. Several studies reported detection of PPV2, PPV3, and PPV4 in Hungary, Romania, Thailand, Japan, and South Africa [18,21,22,23,24]. Moreover, PPV3, PPV4, and PPV7 were found in the United Kingdom, Brazil, and Korea, respectively [25,26,27].

Unlike PPV1, the importance of novel porcine parvoviruses for pig health is poorly understood. These viruses were detected in pigs with different clinical signs, but also in healthy ones [6,10,16,19,20,21,27,28,29,30,31,32]. They were detected in various types of samples from swine, including serum, feces, liver, lungs, heart, spleen, kidney, lymph nodes, tonsils, and aborted fetuses [7,9,10,16,19,20,21,26,27,32,33].

The true role of novel parvoviruses for pig health is difficult to define unequivocally, because they have never been isolated in cell culture and experimental infections have not been performed. However, the significance of any virus can be estimated through the analysis of its circulation in the farms from different regions, countries, types of production, and health status. Unfortunately, the majority of published results on novel porcine parvoviruses were obtained from the analysis of archived samples, sometimes from cases of unexplained disease conditions and routine diagnostic cases of viral and bacterial infections submitted to diagnostic laboratories or that were randomly collected at slaughter [10,16,17,18,19,22,23,33,34]. Until present, there were only two reports published on the systematic analysis of the circulation of different porcine parvoviruses in different farms [19,20]. Detailed information about the on-farm dynamics of porcine parvoviruses circulation, through testing of different diagnostic materials collected from the same populations of animals in a systematic manner, may help to define their biology and epidemiology, as well as to predict their possible interference with the other porcine pathogens.

The present study, performed on samples from 19 Polish pig farms, was aimed to better understand the on-farm prevalence and circulation of PPV1–PPV6, and to assess the diagnostic applicability of testing serum, feces, and oral fluid.

## 2. Materials and Methods

### 2.1. Sample Collection

All samples used in this study were obtained in the framework of a routine surveillance program. The samples were collected cross-sectionally from 19 randomly selected Polish swine farms between 2015 and 2017 (Table S1). The sow herd size ranged from 20 to 3800 and most of the farms produced from farrow to finish. Only Farms WA, BA, B, and C consisted of two sites. In each farm, except Farm AK, vaccination against PPV1 was performed. 

The samples were obtained from randomly selected pigs from different age groups, e.g., from 3, 5, 7, 9, 11, 13, 15, and 17 weeks old or 3, 6, 9, 12, 15, 18, and 21 weeks old (Table S1). From each sampled age group, 4–10 blood samples and fecal swabs were collected. Fecal swabs were obtained from the same pigs as blood (in 3-week-old piglets from Farm KO only serum samples were collected). Oral fluid samples were collected using pieces of a cotton rope, which were hung in each pen for 15–20 min, giving the opportunity to pigs to chew on them. Subsequently, liquid from each piece of rope was squeezed out in a plastic bag, transferred to a plastic tube, and consisted of a pen representative oral fluid sample. In 3-week-old piglets’, oral fluids were collected individually with cotton swabs. Samples were transported to the laboratory and blood samples were centrifuged to obtain sera. All collected samples were stored at −20 °C until usage.

### 2.2. Samples Processing and DNA Extraction

Prior to nucleic acid extraction, thawed fecal swabs from all pigs and oral fluid swabs from 3-week-old piglets were suspended in 1 ml of phosphate-buffered saline (PBS), vortexed for five minutes, and the impurities spun down. Oral fluid samples were left at room temperature to thaw and to allow the larger impurities to sediment prior to supernatants aspiration. The serum and fecal samples, as well as the oral fluids from suckling piglets, were pooled by 4–6 before nucleic acids extraction. The remaining oral fluid samples were tested individually.

Genetic material was extracted from 200 µL of pooled serum, fecal, and oral fluid samples with QIAamp DNA Mini Kit or QIAamp cador Pathogen Mini Kit (Qiagen, Hilden, Germany) according to the manufacturer’s instructions. Nucleic acids were eluted with 100–200 µL of the elution buffer and stored at −20 °C until analysis.

### 2.3. Real-Time PCR Detection of Porcine Parvoviruses

Three duplex real-time PCR assays were performed to detect PPV1 and PPV2, PPV3 and PPV6, PPV4 and PPV5 using previously published primers and probes [9,32,33,35,36] (Table S2). The amplification was carried out on 6000 Rotor Gene (Qiagen, Hilden, Germany) and each reaction of a total volume of 25.0 µL contained 12.5 µL of a reaction mix (SensiFAST Probe No-ROX, Bioline), 2.0 µL of DNA, 1.0 µL of each 10.0 µM primer, and 0.5 µL of each 10.0 µM probe. The side-by-side analysis of selected DNA samples with single and duplex PCR format proved that duplexing of the primers and probes had no negative impact on real-time PCR results. Additionally, the PCR products from positive controls used in each PCR assay were sequenced and the sequence analysis clearly confirmed specificity of PCR amplification. The samples with Ct ≥ 37 (Ct—cycle threshold) were considered as negative.

### 2.4. Statistical Analysis

For the data analysis, the R software version 3.5.1 [37] and the *rms* package version 5.1-3 [38] were used. Differences in PPV1–PPV6 prevalence rates among age groups and sample types were investigated using Fisher’s exact test by pairwise comparisons. Logistic regression analysis was used to determine the probability of PPV1–PPV6 positive results in different diagnostic materials and age groups. A two-tailed *p*-value < 0.05 was set as the statistically significant level.

## 3. Results

### 3.1. Detection Rates of Porcine Parvoviruses in Pig Farms

**Figure 1:**
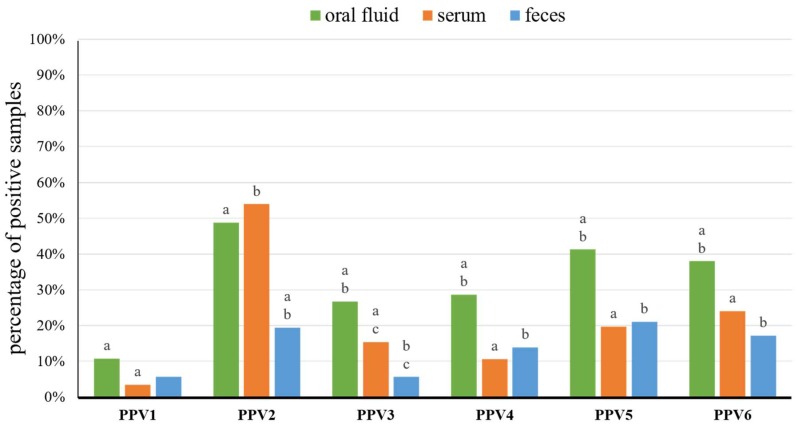
Percentage of porcine parvoviruses 1–6 (PPV1–PPV6) positive samples of oral fluid (*n* = 150), serum (*n* = 254), and feces (*n* = 252). Statistically significant differences (*p* < 0.05, Fisher’s exact test) between diagnostic materials, within grouped bars for each virus, are marked with superscripts on the top of bars in the chart (a, b, and c).

In total, 271 oral fluids, 1244 serum samples, and 1238 fecal samples were collected from 3–21-week-old pigs from 19 pig farms (Table S1). The nucleic acids were extracted from 150 oral fluids, 254 serum pools, and 252 fecal pools (Table S1). Overall, PPV1, PPV2, PPV3, PPV4, PPV5, and PPV6 were detected, in at least one type of sample, in 7, 18, 13, 15, 17, and 16 farms, respectively (Table 1). In the 18 farms, coexistence of three to six porcine parvoviruses (PPVs) was detected in a variety configurations (Table 1). In Farm PR, only PPV2 and PPV4 co-circulated (Table 1). In all samples, from piglets (3–4-week-old), weaners (5–8-week-old), and fatteners (≥9-week-old) taken together, the rates of PPV1–PPV6 detection in oral fluid ranged from 10.7% (PPV1) to 48.7% (PPV2), in sera from 3.5% (PPV1) to 53.9% (PPV2), and in fecal samples from 5.6% (PPV1 and PPV3) to 21.0% (PPV5) (Figure 1, Table 2 and Table S1). The mean real-time PCR Ct values for PPV1–PPV6 positive samples ranged from 29.4 ± 5.0 (PPV6) to 32.3 ± 3.3 (PPV4) in oral fluids, from 22.0 ± 8.2 (PPV6) to 33.1 ± 3.5 (PPV1) in serum pools and from 31.2 ± 4.1 (PPV6) to 32.7 ± 3.0 (PPV3) in fecal pools (Table S3).

### 3.2. Detection Rates of Porcine Parvoviruses in Pigs of Different Age

**Figure 2:**
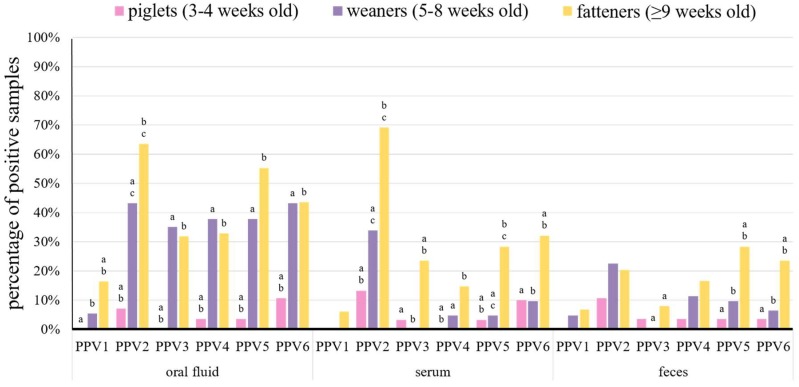
Percentage of porcine parvoviruses 1–6 (PPV1–PPV6) positive samples from piglets (*n* = 86), weaners (*n* = 161), and fatteners (*n* = 405) in different diagnostic materials (oral fluid, serum, and feces). Statistically significant differences (*p* < 0.05, Fisher’s exact test) between age groups in diagnostic materials, within bars grouped for each virus, are marked with superscripts on the top of bars in the chart (a, b, and c).

Suckling piglets positive for at least one PPV species were detected in six farms (Table 1). All PPVs, except for PPV1, were found in this group (Figure 2, Table 1 and Table 2). All types of samples from piglets were positive for PPV2, PPV5, and PPV6, while PPV3 was found only in serum and feces, and PPV4 was present only in oral fluid and feces (Figure 2 and Table 2).

Porcine parvovirus 2 was the most common species in piglets and it was found in 7.1% of oral fluids, 13.3% of serum pools, and 10.7% of fecal pools from this group (Figure 2 and Table 2). The second most frequent virus in piglets was PPV6 and it was detected in 10.7% of oral fluid pools, 10.0% of serum pools, and 3.6% of fecal pools (Figure 2 and Table 2). The remaining viruses were detected in less than 4.0% of each type of tested samples (Figure 2 and Table 2).

In piglets co-circulated PPV3, PPV5, and PPV6 in Farm PB, PPV2, and PPV6 in Farms B and WT, and PPV4 and PPV6 in Farm KU (Table 1). The litters sampled from Farms SU and RO were infected with a single parvovirus, PPV2 or PPV3, respectively (Table 1).

Testing of different diagnostic materials from piglets showed low mean Ct values (Ct ≤ 25.0), suggestive for high virus loads in some individual piglets, included in serum pools positive for PPV2 and PPV5 (Ct = 22.2 ± 4.8 and Ct = 20.4 ± 0.0, respectively) (Figure S1 and Table S3). Mean Ct values between 25.0 and 30.0 were found for PPV2 and PPV6 in positive fecal pools (Figure S1 and Table S3). The remaining positive samples showed mean Ct values ≥ 30.0 suggesting low virus loads (Figure S1 and Table S3).

In weaners, PPVs were present in almost all the farms, except Farm AK (Table 1), and all the PPV species were detected in this group (Figure 2). Most of the PPVs were detected in all types of samples from weaners, only PPV1 was not present in serum, and PPV3 was not detected in serum and feces (Figure 2 and Table 2).

Similar to suckling piglets, PPV2 was the most common species in weaners, which was found in 43.2% of oral fluids, 33.9% of serum pools, and 22.6% of fecal pools (Figure 2 and Table 2). The other PPVs were detected in less than 38.0% of oral fluids (except for PPV6 – 43.2%), 10% of serum, and 12.0% of feces (Figure 2 and Table 2). Overall, all PPVs were detected more often in weaner than in piglet samples, but statistically significant differences (*p* < 0.05) were present in serum samples for PPV2, PPV4, and PPV5 and in oral fluids for almost all PPVs (except for PPV1) (Figure 2).

The weaners from Farms PA, B, C, and SU were positive only for PPV2 (Table 1). In the other farms, all PPV species were detected in this age group, and from two to five PPVs species in a variety of configurations co-circulated (Table 1).

Similar to suckling piglets, most of the mean Ct values in positive samples of different diagnostic materials were high (Ct ≥ 30.0) (Figure S1 and Table S3). The lowest mean Ct values (Ct ≤ 25.0) were found in serum pools positive for PPV4 (Ct = 23.6 ± 2.5), PPV5 (Ct = 25.0 ± 2.1), and PPV6 (Ct = 24.7 ± 11.9) (Figure S1 and Table S3). Mean Ct values, between 25.0 and 30.0, were found for PPV2 positive serum pools (Ct = 27.4 ± 6.2) (Figure S1 and Table S3).

All PPVs were detected in all types of diagnostic materials from fattening pigs (Figure 2). As in younger groups, PPV2 was the most common species in serum pools and oral fluid samples from fatteners (69.1% and 63.5%, respectively) (Figure 2 and Table 2). However, PPV5 was the most prevalent in feces (28.4%) (Figure 2 and Table 2). The other PPVs were detected in less than 56.0% of oral fluids, 33.0% of sera, and 24.0% of feces (Figure 2 and Table 2). The detection rates of PPV1–PPV6 in oral fluids, PPV2–PPV6 in serum pools and PPV3, PPV5, and PPV6 in fecal pools were significantly (*p* < 0.05) higher than in piglets and/or weaners (Figure 2).

In four farms double infection (PPV2 with PPV5 in Farms BO and B; PPV2 with PPV4 in Farms PR and RO) and in three farms triple infection (PPV2, PPV5, and PPV6 in Farms A and SU; PPV1, PPV2, and PPV5 in Farm GO) were detected in fatteners (Table 1). In the remaining farms, from four to six PPVs co-circulated in a variety of configurations (Table 1).

The mean Ct values in positive samples of different diagnostic materials obtained from fatteners were similar to the aforementioned values in younger pigs, and most of them were ≥30.0 (Figure S1 and Table S3). Only PPV6 positive serum pools showed the mean Ct value much below 30.0 (Ct = 21.0 ± 7.3) (Figure S1 and Table S3). Mean Ct values between 25.0 and 30.0 were found in serum pools positive for PPV3, PPV4, and PPV5 (Ct = 29.2 ± 6.1, Ct = 25.7 ± 5.7, and Ct = 25.4 ± 5.8, respectively) (Figure S1 and Table S3).

### 3.3. Probability of PPV1–PPV6 Detection in Different Diagnostic Materials and Age Groups

The real-time PCR results showed that oral fluid was a sample type with the highest detection rates for almost all PPVs, only PPV2 was slightly more prevalent in serum than in oral fluid (53.9% and 48.7%, respectively) (Figure 1 and Table 2). Among the age groups, the highest detection rates were noted in fatteners for all PPV species (Figure 2 and Table 2). Therefore, oral fluid and fatteners were chosen as the reference levels in a logistic regression model to determine the probability of the detection of different PPV species in the variables including different diagnostic materials (levels of variable: oral fluid, serum, feces) and age groups (levels of variable: piglets, weaners, fatteners). The probability of detection, for most PPV species, was the highest in oral fluid (Figure S2). Moreover, the detection probability of most PPVs in serum was decreased by 53.0–74.0%, and in feces by 61.0–80.0%, as compared to oral fluid (Table S4). Only the PPV2 detection probability in serum was 9.0% higher than in oral fluid (Figure S2 and Table S4). Likewise, fatteners showed to be the age group with the highest PPVs detection probability, in comparison with weaners and piglets (Figure S2). Furthermore, the probability of PPV1–PPV6 detection in weaners or piglets was 53.0–74.0% and 84.0–99.9% lower than in fatteners, respectively (Table S4).

## 4. Discussion

Understanding the role of novel porcine parvoviruses in swine health disorders is difficult. In contrast to PPV1, PPV2–PPV6 have never been cultured in vitro, thus challenge experiments could not be performed. Their significance can only be inferred, or speculated, from their detection at the DNA level. However, the detection of such viruses in samples from clinical cases of unknown etiology may lead to incorrect conclusions on their pathogenic role. Thus, studying the patterns of circulation of novel PPVs in different farms may provide more objective data, and contribute to better understanding the ecology of these viruses. For this purpose, our study was performed on 19 Polish farms, from which sera, feces, and oral fluids from groups of pigs at different age were analyzed by real-time PCR for the presence of PPV1–PPV6 DNA. To our knowledge, this is the largest analysis of PPVs circulation in standard pig farms performed to date.

Among all 19 pig farms investigated, PPV1 was found in seven, PPV2 in 18, PPV3 in 13, PPV4 in 15, PPV5 in 17, and PPV6 in 16 farms, in at least one type of sample. This study, as the previous examination of six Polish pig farms [19], and our earlier work on PPV7 (where the virus was detected in all 14 tested farms) [20], indicates that all novel porcine parvoviruses are widely spread in Polish pigs. 

The detection rates from different countries, or in different farms and age groups from the same country may vary, but it has to be stressed that various sampling and testing protocols used in different studies might influence the results. Although, our study involved testing of more farms and samples than most of the previous reports were based on, only pooled samples were tested.

The comparison of the results from our study and the earlier reports on PPV1–PPV6 detection showed some differences. On the one hand, the detection of PPV1, PPV2, and PPV3 in serum pools or PPV3 and PPV4 in feces were similar or slightly different than in previous reports (Table 2) [7,16,21,26,28,34]. On the other hand, the detection rates of PPV4, PPV5, and PPV6 in serum pools and PPV2 and PPV5 in fecal samples were significantly higher than in previous reports (Table 2) [9,10,16,17,19,21,28,33]. The identification of PPV6 in 17.1% of fecal pools is the first report on the detection of this virus in feces. Interestingly, the detection rates of PPV2–PPV6 in feces were significantly lower than those of PPV7, which was present in 39.0% of fecal pools from 14 Polish farms [20].

Over the last years, oral fluid gained global popularity as a welfare friendly and easy to obtain diagnostic sample [39]. However, the detection of PPVs was not described in this type of sample. Our study showed that all PPVs can be readily detected in such material, with the prevalence ranging from 10.7% (PPV1) to 48.7% (PPV2). Thus, oral fluid can be considered as a sample of choice in PCR surveillance of PPVs, as almost all species were detected more often in oral fluid than in serum or feces (except serum for PPV2) (Figure 1). Furthermore, the PPVs detection probability in serum and feces was lower by more than 50.0% in comparison with oral fluid (Figure S2 and Table S4).

Parvoviruses are known to be resistant to disinfection, thus the most likely explanation for the highest detection rates of PPVs in oral fluid is that these viruses contaminate pig housing facilities. However, their presence in fecal samples is more difficult to explain. The detection of their DNA in fecal pools can either indicate current shedding of viruses replicating in alimentary tract by one or more pigs from a given pen, or their passive passage from contaminated pen environment through alimentary tract of non-infected animals. Thus, the detection of PPVs in oral fluids or fecal samples does not necessarily reflect the fact of ongoing infection on a population level, while the results obtained from serum may be more correct in this regard.

The available data on PPVs’ prevalence in suckling piglets is very limited and come from single studies from the USA and Poland, where no parvoviruses were detected in blood [19,33] and only 3.0% of fecal samples were positive for PPV5 [9] in the youngest animals. By contrast, our study showed the presence of almost all PPVs in piglet samples (except PPV1 in all sample types, PPV3 in oral fluids, and PPV4 in serum pools) (Table 2). No samples were collected from lactating sows, from which progeny was examined in this study, thus it is difficult to define if PPVs infected piglets in utero, or from shedding sows in breeding pens, or from insufficiently disinfected environment.

Our study revealed significantly higher detection rates of PPV2 and PPV4 in weaners and fatteners than reported previously (Table 2) [9,10,19,33]. The prevalence of PPV2–PPV6 in serum pools of fatteners was about two to four times higher than previously reported (Table 2) [10,19]. Interestingly, PPV1 viremia was detected only in 9–17 weeks old pigs, in 6.2% of serum pools in this study. The detection rates of PPV2, PPV4, and PPV5 in feces from fatteners were also much higher in our examination (Table 2) [9,33]. On the other hand, the detection rate of PPV5 in weaners, found in this study, was only slightly higher than in previous examination of sera from Polish pigs at the age of 5–8 weeks (Table 2) [19]. Interestingly, only this study showed the presence of PPV4 (4.8%) and PPV6 (9.7%) in weaner serum, and PPV3 (8.0%) and PPV6 (23.5%) in fattener feces.

While in piglets and weaners PPV2 dominated in all types of samples, in fatteners PPV5 was the most prevalent species in feces (28.4%). Still, the most common PPV species in feces from fatteners seems to be PPV7, with the prevalence rate of 56.8% [20].

The fatteners were the age group with significantly (*p* < 0.05) higher prevalence of PPV1–PPV6 in serum than piglets and weaners (Figure 2). In total, PPV detection probability in samples from piglets and weaners was lower by more than 80.0% and 50.0% than in fatteners (Figure S2 and Table S4). Similar differences in the detection rates of PPVs in serum samples, between different age groups of pigs, were observed in three earlier investigations, where PPVs were detected more often in growing and finishing pigs than in piglets [19,20,33]. 

The low prevalence of PPV1 in pigs can be explained by the common vaccination of sows against parvovirosis and the passive immunity of their progeny. However, persistence of maternally derived antibodies may vary significantly, from 9 to 22 weeks [40,41,42,43]. Moreover, Foerster et al. [44] showed that PPV1 can be detected in body excretions, such as feces and nasal discharge, from vaccinated sows from the 9th day post PPV1 inoculation, for at least 49 days. Thus, the detection of PPV1 in low but significant proportions of oral fluids and fecal pools from weaners and fatteners is not surprising, as maternal antibodies may not protect pigs until the end of the fattening period and even vaccinated animals can shed the virus.

The detection of PPV2 in all age groups, in majority of the farms, indicates that this infection commonly starts in piglets or weaners (Figure S3). Infections with PPV3–PPV6 were significantly less frequent in suckling piglets, and in most farms, weaners or even fatteners, were the youngest animals affected (Figure S3). Thus, it can be assumed that passive immunity against PPV3–PPV6 is protective for piglets, but less so against PPV2. Interestingly, this is in contrast to the previous studies which showed relatively high levels of PPV2-specific antibodies in pigs at the age of 2–6 weeks, which were protective against infection [19,34]. Similarly, as in the case of PPV7 [20], infections of PPV2–PPV6 persisted until the late fattening period in most of the positive farms, which suggests the chronic character of these infections (Figure S3). 

Despite some indications that novel porcine parvoviruses may contribute to pig pathologies, strong evidence supporting this is still lacking. Recently, the PPV2 tropism to lymphoid tissue was suggested by Novosel et al. [29], as the virus was found mostly in immature B lymphocytes and/or NK lymphocytes in lungs, which may indicate that PPV2 contributes to the pathogenesis of pneumonia. In this study, we showed that some PPV6 positive serum pools from fatteners had Ct values as low as 10.7. Other serum pools reacted positive for PPV2, PPV3, PPV4, and PPV5 with Ct values of 13.5, 12.0, 17.7, and 13.6, respectively. Such low Ct values are suggestive for high levels of viremia in at least some individual pigs included in these pools. Although, all examined pigs were clinically healthy during the sample collection, it raises a question if intensive replication of PPVs may have a negative impact on pig health, especially in the case of co-infections with other viruses common in pigs, e.g., PCV2 or porcine reproductive and respiratory syndrome virus (PRRSV).

## 5. Conclusions

To date, this is the largest study on the detection patterns of novel porcine parvoviruses performed in a large number of pig farms. Our results confirmed that novel porcine parvoviruses are common in pigs in Poland, but there are important differences in their detection rates between farms, clinical materials, and age groups of pigs. Among all sample types and age groups investigated, PPV detection probability was the highest in oral fluid and fatteners. Porcine parvovirus 2, being at the forefront, was detected in groups of animals of all ages. Detection patterns of PPVs suggest the chronic character of the infection, as these viruses persisted until a late period of fattening. Moreover, real-time PCR results indicated that PPV2, PPV3, PPV5, and PPV6 are likely to cause a high level of viremia in some individual pigs included in serum pools. Although, these infections in the sampled farms had an apparently subclinical course, their role as co-factors in disease syndromes or complexes, caused by the other primary pathogens, cannot be excluded. Further studies are warranted to assess the impact of the novel PPV infections on pig health.

## Figures and Tables

**Table 1:** The detection and coexistence of porcine parvoviruses 1–6 (PPV1–PPV6) in different age groups of pigs (piglets: 3–4 weeks old, weaners: 5–8 weeks old, and fatteners: ≥9 weeks old) in 19 farms. The group of piglets (p), weaners (w) or fatteners (fa) was considered as positive if at least one sample type (oral fluid, serum or feces) was positive for PPV1 –PPV6. The age groups positive for PPV1-PPV6 are marked with symbols: ▲—PPV1, ■—PPV2, □—PPV3, ●—PPV4, ○—PPV5, ♦—PPV6.

Farm ID	PPV1			PPV2			PPV3			PPV4			PPV5			PPV6		
	p	w	fa	p	w	fa	p	w	fa	p	w	fa	p	w	fa	p	w	fa
AK			▲						□			●						♦
GO		▲	▲		■	■					●			○	○		♦	
WA		▲	▲		■	■		□	□		●	●			○		♦	♦
PB			▲		■	■	□	□	□		●	●	○	○	○	♦	♦	♦
PA					■	■			□			●			○			♦
BA					■	■		□	□		●	●		○	○		♦	♦
BO					■	■					●			○	○			
KS						■		□	□						○		♦	♦
KO						■		□	□		●	●		○	○		♦	♦
KU						■		□	□	●	●	●		○	○	♦	♦	♦
A						■					●			○	○			♦
B				■	■	■									○	♦		
C					■	■			□			●			○			
PR					■	■					●	●						
RO		▲				■	□	□			●	●		○			♦	
SU				■	■	■									○			♦
WT				■	■	■		□	□			●		○	○	♦	♦	♦
ZD			▲		■	■			□			●			○		♦	♦
GK			▲		■	■			□						○		♦	♦
No. of positive farms in each age group	0	3	6	3	13	18	2	8	12	1	10	12	1	9	16	4	11	13
Total no. of positive farms	7			18			13			15			17			16		

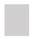
 Samples not collected.

**Table 2:** Comparison of detection rates of porcine parvoviruses 1–6 (PPV1–PPV6), in different age groups of pigs (piglets, weaners, and fatteners) and different materials (of—oral fluid, s—serum, and fe—feces), between this study and earlier reports. Cells marked with “–“ indicate negative results. Light-gray shaded cells correspond to lacking data.

	Age Group (weeks)	PPV1			PPV2			PPV3			PPV4			PPV5			PPV6		
		of	s	fe	of	s	fe	of	s	fe	of	s	fe	of	s	fe	of	s	fe
This study	piglets (3–4)	−	−	−	7.1%	13.3%	10.7%	−	3.3%	3.6%	3.6%	−	3.6%	3.6%	3.3%	3.6%	10.7%	10.0%	3.6%
	weaners (5–8)	5.4%	−	4.8%	43.2%	33.9%	22.6%	35.1%	−	−	37.8%	4.8%	11.3%	37.8%	4.8%	9.7%	43.2%	9.7%	6.5%
	fatteners (≥9)	16.5%	6.2%	6.8%	63.5%	69.1%	20.4%	31.8%	23.5%	8.0%	32.9%	14.8%	16.7%	55.3%	28.4%	28.4%	43.5%	32.1%	23.5%
	TOTAL	10.7%	3.5%	5.6%	48.7%	53.9%	19.4%	26.7%	15.4%	5.6%	28.7%	10.6%	13.9%	41.3%	19.7%	21.0%	38.0%	24.0%	17.1%
Earlier studies [references]	piglets (3–4)															3.0% [9]			
	weaners (5–8)					1.6% [19]	5.1% [33]						1.8% [9]		1.6% [19]				
	fatteners (≥9)					26.6% [19]	10.7% [33]		11.0% [19]			3.5% [19]	3.6% [9]		5.2% [19]	5.4% [9]		8.8–15.5% [10,19]	
	TOTAL		1.1–7.2% [16,21]			5.4–19.0% [19,21] 35.2–55.0% [16,28,34]	6.0–7.6% [21,33]		9.4–14.4% [16,21]	10.0% [7]		2.4–3.3% [16,19,21,28]	1.9% [9] 7.0–18.2% [21,26]		3.4–4.0% [16,19]	2.6% [9]		6.1–15.2% [10,17,19]	

## Supplementary Materials

The following are available online at https://www.mdpi.com/1999-4915/11/5/474/s1, Table S1: Summary of the sampled pig farms characteristics and the prevalence of porcine parvoviruses 1–6 (PPV1–PPV6) in different diagnostic materials collected from 3–21-week-old pigs, Table S2: Primers and probes used for porcine parvoviruses 1–6 (PPV1–PPV6) detection, Table S3: Prevalence and mean Ct values of porcine parvoviruses 1–6 in different age groups of pigs and different diagnostic materials, Table S4: Percentage of increase or decrease of porcine parvoviruses 1–6 (PPV1–PPV6) detection probability in different diagnostic materials and age groups, using the logistic regression model. Figure S1: Boxplots of cycle threshold (Ct) values, from real-time PCR results, for porcine parvoviruses 1–6 (PPV1–PPV6) detection in different samples from piglets, weaners and fatteners, Figure S2: Boxplots of porcine parvoviruses 1–6 (PPV1–PPV6) detection in two variables (diagnostic material and age group) using the logistic regression model, Figure S3: Detection of porcine parvoviruses 1–6 (PPV1–PPV6) in pigs of different age in different diagnostic materials from 19 pig farms.
