# Supplementary material for: Detection Patterns of Porcine Parvovirus (PPV) and Novel Porcine Parvoviruses 2 through 6 (PPV2–PPV6) in Polish Swine Farms
Source: Viruses. 2019 May 24;11(5):474. doi: 10.3390/v11050474 (PMC6563502; doi:10.3390/v11050474)
Supplement: Supplementary file 1 [file viruses-11-00474-s001.zip › viruses-495398-proofreading-supplementary/Figure S1_FINALrev2.docx]

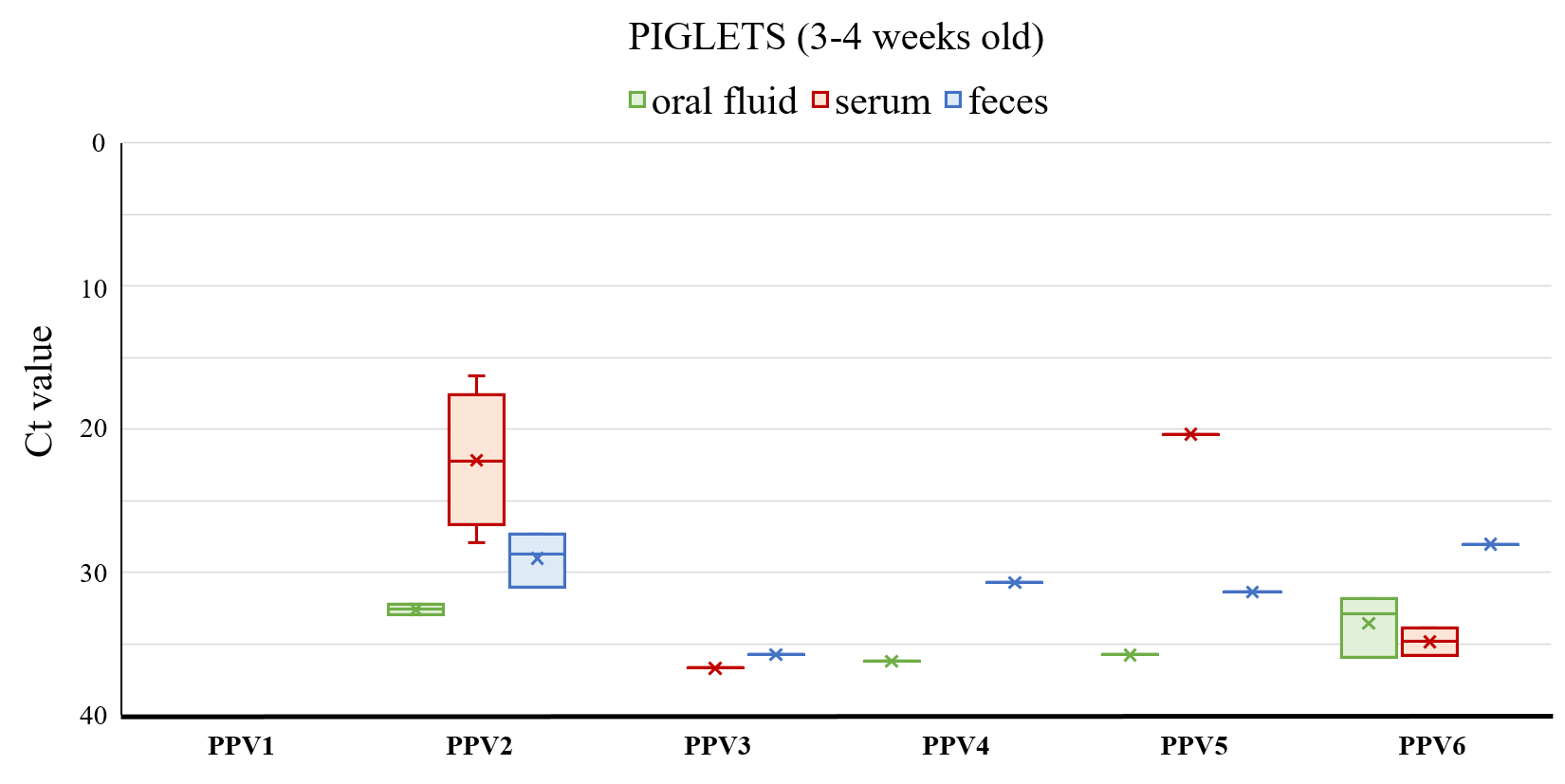


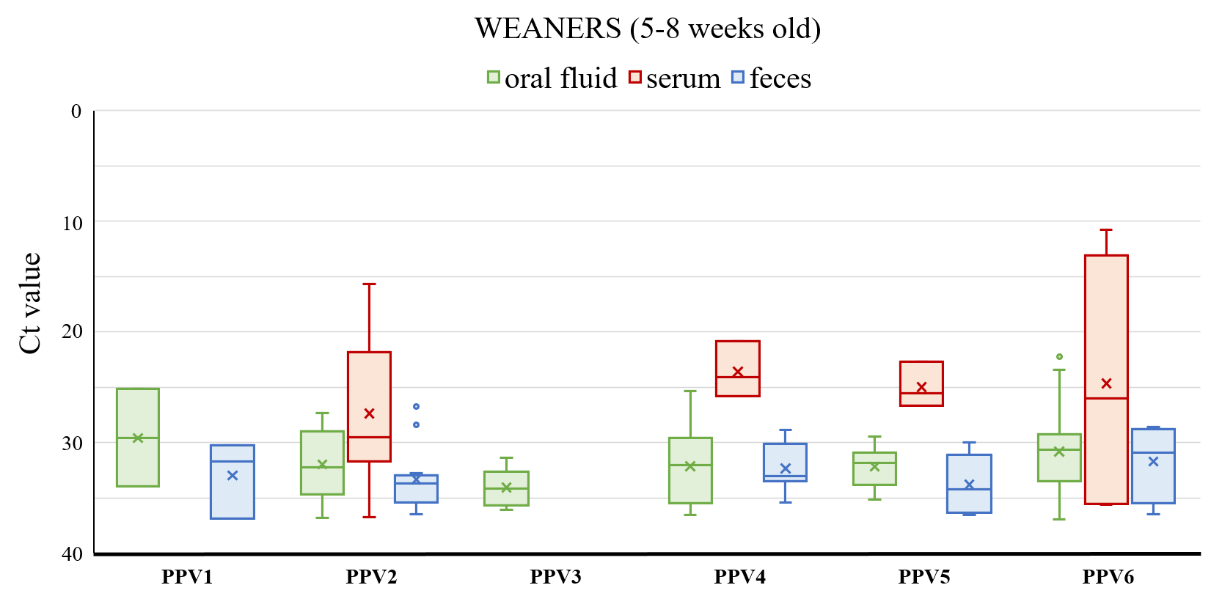


**
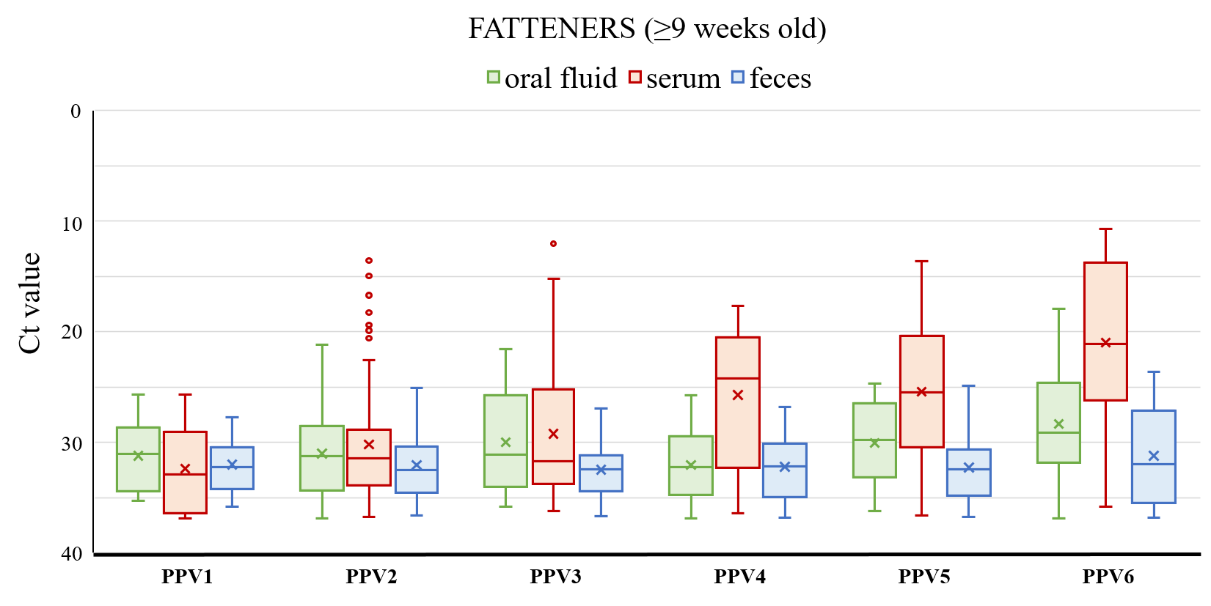
**

**Figure S1.** Boxplots of cycle threshold (Ct) values, from the Real-Time PCR results, for porcine parvoviruses 1-6 (PPV1-PPV6) detection in different samples (oral fluid, serum, feces) from piglets (28 oral fluid, 30 serum and 28 fecal samples), weaners (37 oral fluid, 62 serum and 62 fecal samples) and fatteners (85 oral fluid, 160 serum and 160 fecal samples). In each boxplot horizontal line shows median, cross shows mean, whiskers indicate variability outside the upper and lower quartiles, and any point outside whiskers is considered an outlier.
