# Supplementary material for: Detection Patterns of Porcine Parvovirus (PPV) and Novel Porcine Parvoviruses 2 through 6 (PPV2–PPV6) in Polish Swine Farms
Source: Viruses. 2019 May 24;11(5):474. doi: 10.3390/v11050474 (PMC6563502; doi:10.3390/v11050474)
Supplement: Supplementary file 1 [file viruses-11-00474-s001.zip › viruses-495398-proofreading-supplementary/Figure S2_FINALrev2.docx]

| **VIRUS** | **DIAGNOSTIC MATERIAL** | **AGE GROUP** | |
| --- | --- | --- | --- |
| **PPV1** | 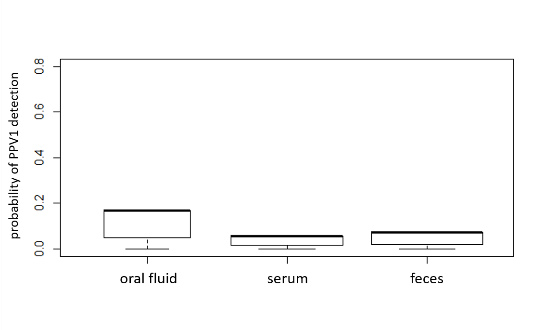 | | 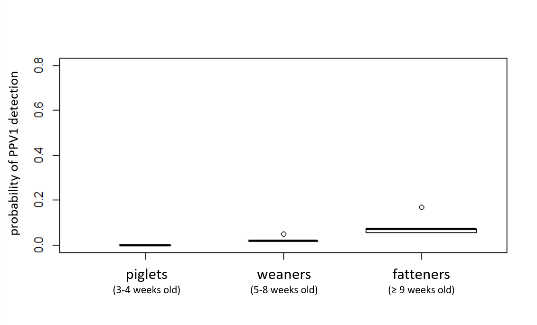 |
| **PPV2** | 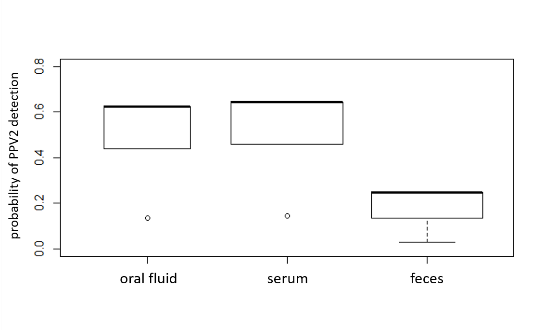 | | 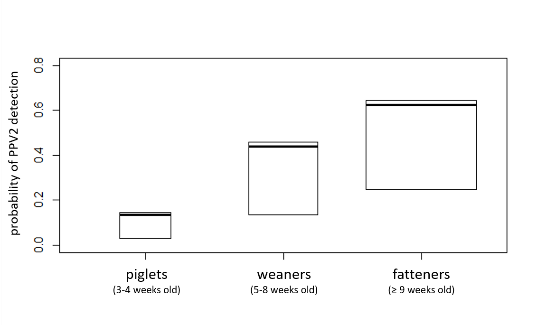 |
| **PPV3** | 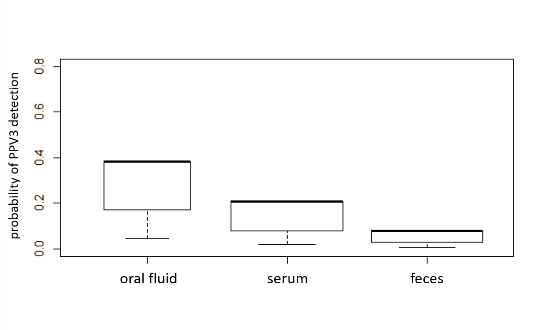 | | 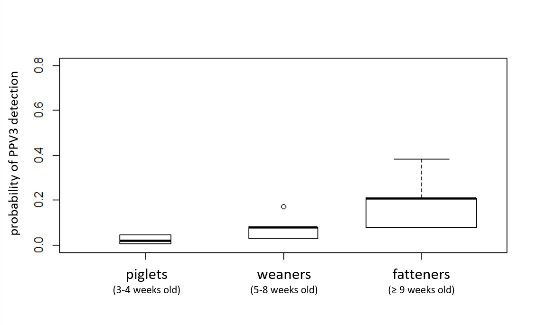 |
| **PPV4** | 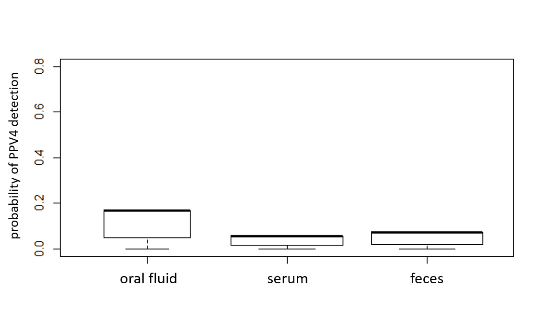 | | 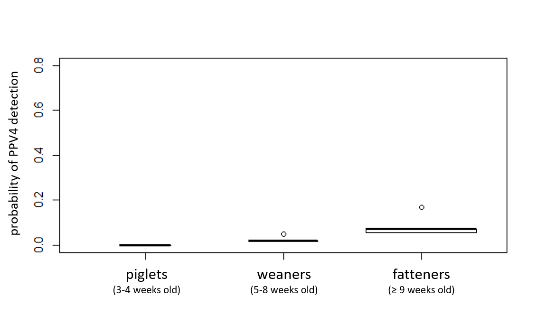 |
| **PPV5** | 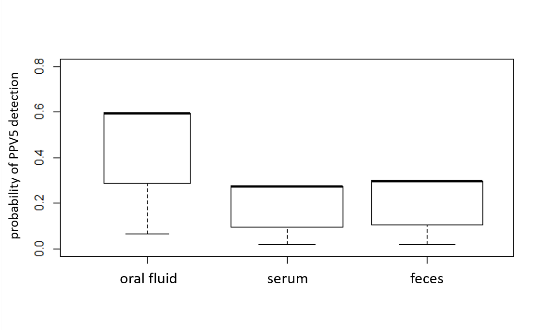 | | 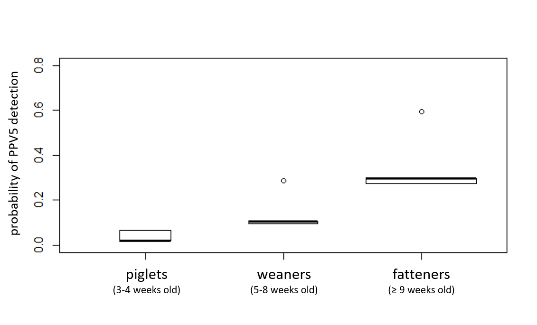 |
| **PPV6** | 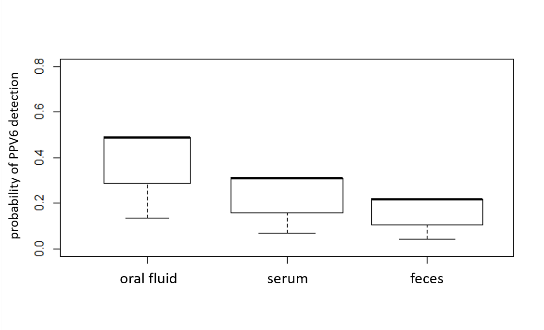 | | 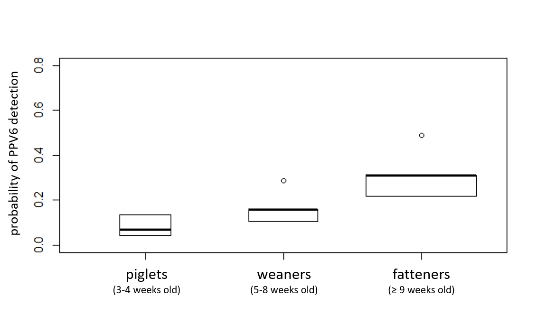 |

**Figure S2.** Boxplots of porcine parvoviruses 1-6 (PPV1-PPV6) detection in two variables (diagnostic material and age group) using the logistic regression model. The levels of first variable were oral fluid (n = 150), serum (n = 254) and feces (n = 252), the levels of second variable were piglets (n = 86), weaners (n = 161) and fatteners (n = 405). The thickness of each boxplot is proportional to the square root from the size of the given group.
