# Supplementary material for: Detection Patterns of Porcine Parvovirus (PPV) and Novel Porcine Parvoviruses 2 through 6 (PPV2–PPV6) in Polish Swine Farms
Source: Viruses. 2019 May 24;11(5):474. doi: 10.3390/v11050474 (PMC6563502; doi:10.3390/v11050474)
Supplement: Supplementary file 1 [file viruses-11-00474-s001.zip › viruses-495398-proofreading-supplementary/Figure S3_FINALrev2.docx]

**Figure S3.** Detection of porcine parvoviruses 1-6 (PPV1-PPV6) in pigs of different age (piglets: 3-4 weeks old, weaners: 5-8 weeks old, fatteners: ≥9 weeks old) from 19 pig farms, in different diagnostic materials (oral fluid – of, serum – s, feces – fe). Positive samples are marked with colored cells (PPV1 – green, PPV2 – yellow, PPV3 – blue, PPV4 – red, PPV5 – purple, PPV6 – orange), negative samples with gray colored cells. The white colored cells shows where samples were not collected. The positive samples are divided into three Ct (cycle threshold) values categories from the Real-Time PCR results (for each virus correspond the same color theme as mentioned above, Ct ≥ 30 - the lightest colored cells, 30 < Ct <25 - medium colored cells, and Ct ≤ 25 – the darkest colored cells).
