# Supplementary material for: Detection Patterns of Porcine Parvovirus (PPV) and Novel Porcine Parvoviruses 2 through 6 (PPV2–PPV6) in Polish Swine Farms
Source: Viruses. 2019 May 24;11(5):474. doi: 10.3390/v11050474 (PMC6563502; doi:10.3390/v11050474)
Supplement: Supplementary file 1 [file viruses-11-00474-s001.zip › viruses-495398-proofreading-supplementary/Table S1_FINALrev2.docx]

| **Farm ID** | **Sow herd size** | **Age in weeks** | **No. of samples**  **(no. of pools)** | | | **Proportion of positive pools** | | | | | | | | | | | | | | | | | |
| --- | --- | --- | --- | --- | --- | --- | --- | --- | --- | --- | --- | --- | --- | --- | --- | --- | --- | --- | --- | --- | --- | --- | --- |
|  |  |  |  |  |  | **PPV1** | | | **PPV2** | | | **PPV43** | | | **PPV4** | | | **PPV5** | | | **PPV6** | | |
|  |  |  | **of** | **s** | **fe** | **of** | **s** | **fe** | **of** | **s** | **fe** | **of** | **s** | **fe** | **of** | **s** | **fe** | **of** | **s** | **fe** | **of** | **s** | **fe** |
| AK | 20 | 3-17 | 35  (11) | 73 (16) | 73 (16) | 2/11 | 2/16 | 2/16 | - | - | - | 5/11 | 7/16 | 1/16 | 4/11 | 3/16 | 3/16 | - | - | - | 2/11 | 2/16 | 2/16 |
| GO | 70 | 3-18 | 14  (8) | 60 (12) | 60 (12) | 3/8 | - | - | 3/8 | 6/12 | - | - | - | - | 1/8 | - | - | 4/8 | 5/12 | 4/12 | 1/8 | - | - |
| WA | 200 | 3-15 | 14  (6) | 50 (10) | 50 (10) | 4/6 | 3/10 | 4/10 | 4/6 | 7/10 | 4/10 | 4/6 | 4/10 | - | 4/6 | 1/10 | 3/10 | 3/6 | 1/10 | 4/10 | 4/6 | 7/10 | 5/10 |
| PB | 2300 | 3-17 | 17  (9) | 70 (14) | 70 (14) | 2/9 | 1/14 | 2/14 | 5/9 | 8/14 | 6/14 | 3/9 | 3/14 | 3/14 | 6/9 | 2/14 | 4/14 | 8/9 | 5/14 | 6/14 | 6/9 | 2/14 | 4/14 |
| PA | 1000 | 3-17 | 15  (8) | 80 (16) | 80 (16) | - | - | - | 5/8 | 11/16 | 4/16 | 5/8 | 6/16 | 2/16 | 1/8 | - | - | 5/8 | 3/16 | 4/16 | 5/8 | 8/16 | 4/16 |
| BA | 600 | 3-17 | 18  (10) | 80 (16) | 80 (16) | - | - | - | 7/10 | 10/16 | 3/16 | 4/10 | 5/16 | 3/16 | 3/10 | - | 2/16 | 3/10 | 3/16 | 2/16 | 4/10 | 3/16 | 2/16 |
| BO | 60 | 5-17 | 6  (5) | 50 (10) | 50 (10) | - | - | - | 2/5 | 5/10 | - | - | - | - | 1/5 | - | - | 4/5 | 4/10 | 2/10 | - | - | - |
| KS | 180 | 6-16 | 7  (7) | 60 (12) | 60 (12) | - | - | - | 3/7 | 6/12 | 4/12 | 2/7 | - | 1/12 | - | - | - | 3/7 | 5/12 | 2/12 | 6/7 | 8/12 | 4/12 |
| KO | 60 | 3-20 | 6  (6) | 66 (14) | 60 (12) | - | - | - | 1/6 | 6/14 | - | 4/6 | - | - | 4/6 | 6/14 | 6/12 | 4/6 | 3/14 | 8/12 | 5/6 | 7/14 | 8/12 |
| KU | 65 | 3-18 | 15  (7) | 60 (12) | 60 (12) | - | - | - | 1/7 | 3/12 | 2/12 | 2/7 | - | - | 6/7 | 5/12 | 6/12 | 6/7 | 2/12 | 2/12 | 7/7 | 4/12 | 4/12 |
| A | 240 | 3-21 | 14  (8) | 56 (14) | 56 (14) | - | - | - | 3/8 | 5/14 | - | - | - | - | 1/8 | - | - | 2/8 | - | - | 2/8 | 2/14 | 2/14 |
| B | 220 | 3-21 | 16  (8) | 70 (14) | 70 (14) | - | - | - | 2/8 | 8/14 | 2/14 | - | - | - | - | - | - | 4/8 | 5/14 | 4/14 | - | 2/14 | - |
| C | 220 | 6-21 | 6  (6) | 60 (12) | 60 (12) | - | - | - | 4/6 | 9/12 | 3/12 | - | 5/12 | - | 1/6 | - | - | 1/6 | 1/12 | - | - | - | - |
| PR | 390 | 3-17 | 17  (10) | 80 (16) | 80 (16) | - | - | - | 7/10 | 11/16 | 5/16 | - | - | - | 4/10 | 6/16 | 5/16 | - | - | - | - | - | - |
| RO | 800 | 3-15 | 15  (8) | 70 (14) | 70 (14) | - | - | 1/14 | 2/8 | 5/14 | 3/14 | 1/8 | 1/14 | - | 3/8 | - | - | 2/8 | - | 2/14 | 2/8 | - | - |
| SU | 100 | 4-19 | 14  (6) | 60 (12) | 60 (12) | - | - | - | 3/6 | 10/12 | 1/12 | - | - | - | - | - | - | 3/6 | 2/12 | 6/12 | - | 1/12 | - |
| WT | 650 | 3-17 | 19  (11) | 60 (12) | 60 (12) | - | - | - | 10/11 | 10/12 | 3/12 | 9/11 | 4/12 | 2/12 | 2/11 | 2/12 | 3/12 | 6/11 | 4/12 | 1/12 | 7/11 | 7/12 | 4/12 |
| ZD | 90 | 5-16 | 6  (7) | 60 (12) | 60 (12) | 3/7 | 3/12 | 3/12 | 5/7 | 6/12 | 2/12 | - | 1/12 | - | 2/7 | 2/12 | 1/12 | 2/7 | 3/12 | 2/12 | 5/7 | 7/12 | 4/12 |
| GK | 3800 | 3-17 | 17  (9) | 79 (16) | 79 (16) | 2/9 | 1/16 | 2/16 | 6/9 | 11/16 | 7/16 | 1/9 | 3/16 | 2/16 | - | - | - | 3/9 | 4/16 | 4/16 | 1/9 | 1/16 | - |
| **TOTAL** | | | 271 (150) | 1244 (254) | 1238 (252) | 16/  150 | 9/  254 | 14/  252 | 73/  150 | 137/  254 | 49/  252 | 40/  150 | 39/  254 | 14/  252 | 43/  150 | 27/  254 | 35/  252 | 62/  150 | 50/  254 | 53/  252 | 57/  150 | 61/  254 | 43/  252 |
|  |  |  | **Percentage** | | | 10.7% | 3.5% | 5.6% | 48.7% | 53.9% | 19.4% | 26.7% | 15.4% | 5.6% | 28.7% | 10.6% | 13.9% | 41.3% | 19.7% | 21.0% | 38.0% | 24.0% | 17.1% |

**Table S1.** Summary of the sampled pig farms characteristics and the prevalence of porcine parvoviruses 1-6 (PPV1-PPV6) in different diagnostic materials collected from 3-21-week-old pigs in each farm. The percentage of Real Time PCR positive oral fluid (of), serum (s) and faecal (fe) pools are showed for PPV1-PPV6 in each farm. Negative results are marked with “-“. Dark grey cells show ≥50.0%, medium grey cells show 25.0-50.0% and light grey cells show ≤25.0% of positive serum, faecal or oral fluid pools.
