# Supplementary material for: Detection Patterns of Porcine Parvovirus (PPV) and Novel Porcine Parvoviruses 2 through 6 (PPV2–PPV6) in Polish Swine Farms
Source: Viruses. 2019 May 24;11(5):474. doi: 10.3390/v11050474 (PMC6563502; doi:10.3390/v11050474)
Supplement: Supplementary file 1 [file viruses-11-00474-s001.zip › viruses-495398-proofreading-supplementary/Table S2_FINALrev2.docx]

Table S2. Primers and probes used for the porcine parvoviruses 1-6 (PPV1-PPV6).

| **Primers/probes** | **Sequence (5’-3’)** | **References** |
| --- | --- | --- |
| PPV1F | CAGAATCAGCAACCTCACCA | Opriessnig et al., 2011 [35] |
| PPV1R | GCTGCTGGTGTGTATGGAAG |  |
| PPV1-Probe | FAM-5’-TGCAAGCTTAATGGTCGCACTAGACA-3’-BHQ |  |
| PPV2F | TACTGAGCCCTAAGACTGACTACAAGC | Xiao et al., 2013 [33] |
| PPV2R | GTTTGTCTCGTTGTTCGTCTGATG |  |
| PPV2-Probe | HEX-5’-AACTGCTACATGAACCACTTTACCCCSTC-3’-BHQ |  |
| PPV3F | CAYGAYGAACGGTACGATGAAAT | Xiao et al., 2012 [32] |
| PPV3R | GCGGTAAAACCTGTGAWAWTTGAAC |  |
| PPV3-Probe | HEX-5’-TAGGTTGATGAATAAGGAGATAGAGAGGGCGG-3’-BHQ |  |
| PPV4F | GCATTGGTGTGTGTCTGTGTCC* | Xiao et al., 2013 [10] |
| PPV4R | GTGGCACATTTGTACATGGGAG* |  |
| PPV4-Probe | FAM-5’-CTCCGCGGGATGTGCTTACAATTTTCA-3’-BHQ |  |
| PPV5F | GCATTGGTGTGTGTCTGTGTCC* | Xiao et al., 2013 [9] |
| PPV5R | GTGGCACATTTGTACATGGGAG* |  |
| PPV5-Probe | HEX-5’-ACTTTGGTGTTGAGGGACTTAGCTTTTTTGTAC-3’-BHQ |  |
| PPV6F | GGCTTCATAATCCCTCCAAAACCT | Cui et al., 2017 [36] |
| PPV6R | GCTCATCTTCCTCTTGTTTCTCCTG |  |
| PPV6-Probe | FAM-5’-CCTCCTCCTCCTCCCTCTCCAATTCCT-3’-BHQ |  |
