# Supplementary material for: Detection Patterns of Porcine Parvovirus (PPV) and Novel Porcine Parvoviruses 2 through 6 (PPV2–PPV6) in Polish Swine Farms
Source: Viruses. 2019 May 24;11(5):474. doi: 10.3390/v11050474 (PMC6563502; doi:10.3390/v11050474)
Supplement: Supplementary file 1 [file viruses-11-00474-s001.zip › viruses-495398-proofreading-supplementary/Table S3_FINALrev2.docx]

| Age group (weeks of age) | No. of pools | | | PPV1 | | | PPV2 | | | PPV3 | | | PPV4 | | | PPV5 | | | PPV6 | | | prevalence % (proportion) of positive samples at least for one PPVs species | | |
| --- | --- | --- | --- | --- | --- | --- | --- | --- | --- | --- | --- | --- | --- | --- | --- | --- | --- | --- | --- | --- | --- | --- | --- | --- |
|  | of | s | fe | of | s | fe | of | s | fe | of | s | fe | of | s | fe | of | s | fe | of | s | fe | of | s | fe |
| Piglets (3-4) | 28 | 30 | 28 | prevalence % (proportion) | | | | | | | | | | | | | | | | | | 21.4%  (6/28) | 23.3%  (7/30) | 17.8%  (5/28) |
|  |  |  |  | 0.0%  (0/28)^a^ | 0.0%  (0/30) | 0.0%  (0/28) | 7.1%  (2/28) | 13.3%  (4/30) | 10.7%  (3/28) | 0.0%  (0/28) | 3.3%  (1/30) | 3.6%  (1/28) | 3.6%  (1/28) | 0.0%  (0/30) | 3.6%  (1/28) | 3.6%  (1/28) | 3.3%  (1/30) | 3.6%  (1/28) | 10.7%  (3/28) | 10.0%  (3/30) | 3.6%  (1/28) |  |  |  |
|  |  |  |  | m Ct ±SD (range) | | | | | | | | | | | | | | | | | |  |  |  |
|  |  |  |  | 0.0 ±0.0  (0.0-0.0) | 0.0 ±0.0  (0.0-0.0) | 0.0 ±0.0  (0.0-0.0) | 32.6 ±0.5  (32.2-33.0) | 22.2 ±4.8  (16.3-28.0) | 29.0 ±1.9  (27.3-31.0) | 0.0 ±0.0  (0.0-0.0) | 36.7 ±0.0  (36.7) | 35.8 ±0.0  (35.8) | 36.2 ±0.0  (36.2) | 0.0 ±0.0  (0.0-0.0) | 30.7 ±0.0  (30.7) | 35.8 ±0.0  (35.8) | 20.4 ±0.0  (20.4) | 31.4 ±0.0  (31.4) | 33.6 ±2.2  (31.8-36.0) | 34.8 ±1.0  (33.9-35.8) | 28.0 ±0.0  (28.0) |  |  |  |
| Weaners (5-8) | 37 | 62 | 62 | prevalence % (proportion) | | | | | | | | | | | | | | | | | | 83.8%  (31/37) | 46.8%  (29/62) | 38.7%  (24/62) |
|  |  |  |  | 5.4%  2/37 | 0.0%  (0/62) | 4.8%  (3/62) | 43.2%  (16/37) | 33.9%  (21/62) | 22.6%  (14/62) | 35.1%  (13/37) | 0.0%  (0/62) | 0.0%  (0/62) | 37.8%  (14/37) | 4.8%  (3/62) | 11.3%  (7/62) | 37.8%  (14/37) | 4.8%  (3/62) | 9.7%  (6/62) | 43.2%  (16/37) | 9.7%  (6/62) | 6.5%  (4/62) |  |  |  |
|  |  |  |  | m Ct ±SD (range) | | | | | | | | | | | | | | | | | |  |  |  |
|  |  |  |  | 29.6 ±6.2  (25.2-34.0) | 0.0 ±0.0  (0.0-0.0) | 33.0 ±3.5  (30.3-36.9) | 32.0 ±3.0  (27.4-36.8) | 27.4 ±6.2  (15.7-36.7) | 33.3 ±1.5  (26.7-36.5) | 34.0 ±1.5  31.4-36.1) | 0.0 ±0.0  (0.0-0.0) | 0.0 ±0.0  (0.0-0.0)  . | 32.2 ±3.3  (25.3-36.5) | 23.6 ±2.5  (20.8-25.8) | 32.4 ±2.2  (28.9-35.4) | 32.2 ±1.7  (29.5-35.2) | 25.0 ±2.1  (22.7-26.7) | 32.4 ±2.7  (30.0-36.6) | 30.8 ±4.2  (22.2-36.9) | 24.7 ±11.9  (10.8-35.6) | 31.7 ±3.6  (28.6-36.5) |  |  |  |
| Fatteners (>9) | 85 | 160 | 160 | prevalence % (proportion) | | | | | | | | | | | | | | | | | | 91.7%  (78/85) | 87.7%  (142/162) | 58.1%  (93/162) |
|  |  |  |  | 16.5%  (14/85) | 6.2%  (10/162) | 6.8%  (11/162) | 63.5%  (54/85) | 69.1%  (112/162) | 20.4%  (33/162) | 31.8%  (27/85) | 23.5%  (38/162) | 8.0%  (13/162) | 32.9%  (28/85) | 14.8%  (24/162) | 16.7%  (27/162) | 55.3%  (47/85) | 28.4%  (46/162) | 28.4%  (46/162) | 43.5%  (37/85) | 32.1%  (52/162) | 23.5%  (38/162) |  |  |  |
|  |  |  |  | m Ct ±SD (range) | | | | | | | | | | | | | | | | | |  |  |  |
|  |  |  |  | 31.2 ±3.2  (25.7-35.3) | 32.4 ±4.0  (25.7-36.9) | 32.0 ±2.4  (27.8-35.9) | 31.0 ±3.7  (21.2-36.9) | 30.3 ±5.3  (13.5-36.8) | 31.1 ±6.3  (25.1-36.6) | 30.0 ±4.6  (21.6-35.8) | 29.2 ±6.1  (12.0-36.2) | 32.5 ±2.5  (26.9-36.7) | 32.0 ±3.3  (25.7-36.9) | 25.7 ±5.7  (17.7-36.4) | 32.2 ±2.9  (26.8-36.8) | 30.0 ±3.6  (24.7-36.2) | 25.4 ±5.8  (13.6-36.6) | 32.3 ±3.0  (24.9-36.8) | 28.3 ±5.3  (17.9-36.9) | 21.0 ±7.3  (10.7-35.9) | 31.2 ±4.2  (23.6-36.8) |  |  |  |
| Total | | | | prevalence % (proportion) | | | | | | | | | | | | | | | | | |  | | |
|  |  |  |  | 10.7%  (16/150) | 3.5%  (9/254) | 5.6%  (14/252) | 48.7%  (73/150) | 53.9%  (137/254) | 19.4%  (49/252) | 26.7%  (40/150) | 15.4%  (39/254) | 5.6%  (14/252) | 28.7%  (43/150) | 10.6%  (27/254) | 13.9%  (35/252) | 41.3%  (62/150) | 19.7%  (50/254) | 21.0%  (53/252) | 38.0%  (57/150) | 24.0%  (61/254) | 17.1%  (43/252) |  |  |  |
|  |  |  |  | m Ct ±SD (range) | | | | | | | | | | | | | | | | | |  |  |  |
|  |  |  |  | 31.0 ±3.4 (25.2-35.3) | 33.1 ±3.5 (28.0-36.9) | 32.2 ±2.6 (27.8-36.9) | 31.2 ±3.5 (21.2-36.9) | 29.6 ±5.6 (13.5-36.8) | 32.2 ±3.0 (25.1-36.6) | 31.3 ±4.3 (21.6-36.1) | 29.4 ±6.1 (12.0-36.7) | 32.7 ±3.0 (26.9-36.7) | 32.2 ±3.3 (25.3-36.9) | 25.5 ±5.4 (17.7-36.4) | 32.2 ±2.7 (26.8-36.8) | 30.6 ±3.4 (24.7-36.2) | 25.3 ±5.6 (13.6-36.6) | 32.4 ±2.9 (24.9-36.8) | 29.4 ±5.0 (17.9-36.9) | 22.0 ±8.2 (10.7-36.6) | 31.2 ±4.1  (23.6-36.8) |  |  |  |

**Table S3.** Percentage and proportion of porcine parvoviruses 1-6 (PPV1-PPV6) positive samples in different age groups of pigs (piglets, weaners and fatteners) and different materials (of – oral fluid, s – serum pools and fe – fecal pools) from 19 pig farms. Additionally, overall percentage of positive samples at least for one PPV species in each age group is presented.
