# Supplementary material for: Detection Patterns of Porcine Parvovirus (PPV) and Novel Porcine Parvoviruses 2 through 6 (PPV2–PPV6) in Polish Swine Farms
Source: Viruses. 2019 May 24;11(5):474. doi: 10.3390/v11050474 (PMC6563502; doi:10.3390/v11050474)
Supplement: Supplementary file 1 [file viruses-11-00474-s001.zip › viruses-495398-proofreading-supplementary/Table S4_FINALrev2.docx]

**Table S4.** Percentage of increase or decrease of porcine parvoviruses 1-6 (PPV1-PPV6) detection probability in two variables (different diagnostic materials and age groups) using the logistic regression model. The levels of first variable were oral fluid (n = 150), serum (n = 254) and feces (n = 252), the levels of second variable were piglets (n = 86), weaners (n = 161) and fatteners (n = 405). The reference level in each variable is marked with light gray shading. The percentages of increase or decrease of detection probability are the results from the exponentiated coefficients of predictors obtained from logistic regression analysis. For each variable statistical significance was showed for each parvovirus species.

| Variable | Level | percentage of increase (↑) or decrease (↓) of detection probability from reference level | | | | | |
| --- | --- | --- | --- | --- | --- | --- | --- |
|  |  | PPV1 | PPV2 | PPV3 | PPV4 | PPV5 | PPV6 |
| diagnostic material | oral fluid | REFERENCE LEVEL | | | | | |
|  | serum | ↓ 70.0%** | ↑ 9.0% | ↓ 70.0%** | ↓ 70.0%** | ↓ 74.0%*** | ↓ 53.0%** |
|  | feces | ↓ 61.0%* | ↓ 80.0%*** | ↓ 61.0%*** | ↓ 61.0%* | ↓ 71.0%*** | ↓ 71.0%*** |
| age group | fatteners | REFERENCE LEVEL | | | | | |
|  | weaners | ↓ 74.0%* | ↓ 53.0%*** | ↓ 67.0%*** | ↓ 74.0%* | ↓ 72.0%*** | ↓ 58.0%*** |
|  | piglets | ↓ 99.9% | ↓ 91.0%*** | ↓ 92.0%*** | ↓ 99.9% | ↓ 95.0%*** | ↓ 84.0%*** |

Significant codes:

p > 0.05 – no asterisk

p < 0.01 - *

p < 0.001 - **

p ≈ 0 - ***

↑ - increase of probability from the reference level

↓ - decrease of probability from the reference level
